# Overexpression of circulating CD38+ NK cells in colorectal cancer was associated with lymph node metastasis and poor prognosis

**DOI:** 10.3389/fonc.2024.1309785

**Published:** 2024-02-23

**Authors:** Xueling Wang, Haoran Li, Huixian Chen, Kehua Fang, Xiaotian Chang

**Affiliations:** ^1^ Center for Clinical Research, The Affiliated Hospital of Qingdao University, Qingdao, China; ^2^ Department of Hepatobiliary and Pancreatic Surgery, The Affiliated Hospital of Qingdao University, Qingdao, China; ^3^ Clinical Laboratory, The Affiliated Hospital of Qingdao University, Qingdao, China

**Keywords:** CD38+ NK cells, colorectal cancer, lymph node metastasis, prognosis, marker

## Abstract

**Introduction:**

Lymph node metastasis (LNM) is a critical prognostic factor for colorectal cancer (CRC). Due to the potential influence of immune system on CRC progression, investigation into lymphocyte subsets as clinical markers has gained attention. The objective of this study was to assess the capability of lymphocyte subsets in evaluating the lymph node status and prognosis of CRC.

**Methods:**

Lymphocyte subsets, including T cells (CD3+), natural killer cells (NK, CD3- CD56+), natural killer-like T cells (NK-like T, CD3+ CD56+), CD38+ NK cells (CD3- CD56+ CD38+) and CD38+ NK-like T cells (CD3+ CD56+ CD38+), were detected by flow cytometry. Univariate and multivariate analyses were used to assess the risk factors of LNM. The prognostic role of parameters was evaluated by survival analysis.

**Results:**

The proportion of CD38+ NK cells within the NK cell population was significantly higher in LNM-positive patients (*p <*0.0001). However, no significant differences were observed in the proportions of other lymphocyte subsets. Poorer histologic grade (odds ratio [OR] =4.76, *p* =0.03), lymphovascular invasion (LVI) (OR =22.38, *p <*0.01), and CD38+ NK cells (high) (OR =4.54, *p <*0.01) were identified as independent risk factors for LNM. Furthermore, high proportion of CD38+ NK cells was associated with poor prognosis of CRC patients (HR=2.37, *p* =0.03).

**Conclusions:**

It was demonstrated that the proportion of CD38+ NK cells was a marker overexpressed in LNM-positive patients compared with LNM-negative patients. Moreover, an elevated proportion of CD38+ NK cells is a risk factor for LNM and poor prognosis in CRC.

## Introduction

Colorectal cancer (CRC) is one of the most common cancers worldwide, ranking third in terms of incidence and second in terms of mortality among all cancers (1). In CRC, the lymph node status is associated with prognosis and affects clinical treatment decisions. For example, patients with LNM identified during preoperative assessment are typically advocated for neoadjuvant chemotherapy (2). For patients with lesion invasion depth limited to the submucosa (cT1), endoscopic treatment is applicable only when the possibility of LNM is negligible (3). Although previous studies have indicated that pathological factors such as lymphovascular invasion (LVI) can effectively predict LNM in CRC patients (4), these indicators cannot predict lymph node status preoperatively.

Natural killer cells (NK cells) are integral component of the innate immune system and possess cellular cytotoxicity, making them effective immune cells against various threats (5). NK cells are TCR/CD3 complex-negative and are phenotypically defined by the expression of CD56 (6, 7). They exhibit a diverse range of functions that encompass immunoregulatory activities and immune responses against tumor cells and viral infections (8, 9). NK cells play a crucial role in the diagnosis and prognosis of diseases. Previous studies found the circulating CD3- CD56+ CD16+ NK cells were decreased in coronary artery disease (10). In the tumor microenvironment of papillary thyroid cancer, CD3- CD16- CD56bright NK cells were highly expressed (7). The proportion of circulating CD3- CD56+ CD16+ NK cells to lymphocytes was negatively correlated with the occurrence of CRC (11). Additionally, Cui et al. (12) discovered a negative correlation between circulating CD3- CD56+ CD16+ NK cells and the prognosis of CRC. CD38+ NK (CD3- CD56+ CD38+) cell was a subtype of NK cells. Morandi et al. (13) reported that NK cells generate adenosine through a CD38-mediated pathway, which serves as a regulatory mechanism to suppress the proliferation of CD4+ T cell. This suggested that the expression of CD38 on NK cells might had an immunosuppressive role. Indeed, the role of CD38+ NK cells in CRC remains unclear. In this study, flow cytometry was used to detected the proportions of lymphocyte subsets in the peripheral blood of CRC patients before initial treatment. The relationship between CD38+ NK cells and lymph node status was detected, and the risk factors for LNM in CRC patients was identified. In addition, utilizing the follow-up data, we evaluated the factors associated with adverse prognosis.

## Materials and methods

### Blood sample collection

**Figure 1:**
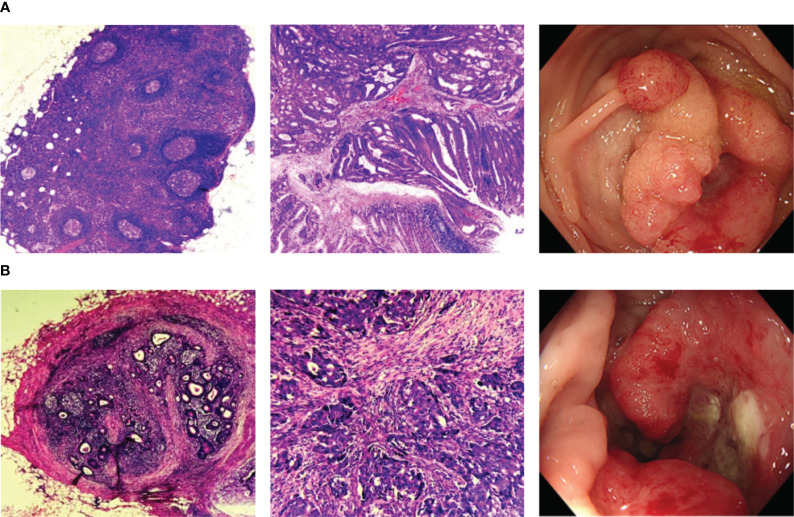
Representative histopathologic and endoscopic images. **(A)** Histopathologic and endoscopic images of CRC patients without LNM; **(B)** Histopathologic and endoscopic images of CRC patients with LNM. CRC, colorectal cancer; LNM, lymph node metastasis.

Between March 2021 and August 2023, a total of 225 blood samples from CRC patients were collected. After excluding duplicates and patients with a history of tumor treatment and/or other neoplastic diseases, 165 patients were finally included in this study. All the enrolled patients were newly diagnosed and had not received any treatment, including surgery, radiation therapy, chemotherapy, and neoadjuvant chemoradiotherapy, at the time of blood sample collection. The blood samples were obtained by laboratory physicians at our hospital. The collection of samples followed standardized procedures and doctor’s prescriptions. Among all the participants, 72 patients were LNM-positive while 93 patients were LNM-negative, as confirmed by endoscopy and postoperative pathology (**Figure 1**). The design of this study adhered to the Declaration of Helsinki of the World Medical Association. The research protocol received approval from the Ethics Committee of the Affiliated Hospital of Qingdao University (QYFYWZLL28002), and all patients provided written informed consent prior to participation.

### Detection of lymphocyte subsets in peripheral blood

600μl red blood cell lysis buffer (Solarbio, China) was added to 200μl fresh peripheral blood and lysed for 2h at 4°C. After centrifugation, the pellets were resuspended by adding 100μl PBS. Antibodies including FITC anti-human CD3 (Biolegend), PE anti-human CD56 (Biolegend) and APC anti-human CD38 (BD Biosciences, USA) were added to the mixture and incubated for 30min at room temperature under light. After centrifugation, the pellets were resuspended with 300ul PBS. Then, T cells (CD3+), NK cells (CD3- CD56+), natural killer-like T (NK-like T) cells (CD3+ CD56+), CD38+ NK (CD3- CD56+ CD38+) and CD38+ NK-like T cells (CD3+ CD56+ CD38+) were detected by flow cytometry (Agilent, USA). CD38 fluorescence-minus-one (FMO) was used as control.

### Data collection

Based on the treatment guidelines set by the Japanese Society for Cancer of the Colon and Rectum (JSCCR), the enrolled patients subsequently underwent colon/rectal resection with lymph node dissection. The histologic slides collected from the procedure were then independently evaluated by two proficient pathologists.

According to the TNM staging classification from the American Joint Committee on Cancer (AJCC) and the Union for International Cancer Control (UICC), the extent of tumor invasion can be categorized into four grades: T1 (no invasion beyond the submucosa), T2 (invasion into the muscularis propria), T3 (invasion reaching the subserosa), and T4 (invasion into the visceral peritoneum or adjacent organs or structures). In addition, tumors were categorized into well-differentiated, moderately-differentiated, and poorly-differentiated adenocarcinomas, as well as mucinous carcinoma or signet ring cell type, according to the most predominant histological feature (14). LVI was assessed using the D2-40 antibody (Dako, Denmark). Perineural invasion was determined by detecting the presence of the S100 protein.

Other clinical and histopathological informations of all patients were collected, including gender, age, presence of ulcers, body mass index (BMI), carcinoembryonic antigen (CEA), location, tumor size and neoadjuvant therapy.

### Statistical analysis

The data analysis was conducted using GraphPad Prism 8.3.0 and SPSS statistical software (version 22.0). For quantitative data with a normal distribution, t-test was employed for analysis, otherwise, the Mann-Whitney U test was utilized. Receiver operating characteristic (ROC) curve analysis was performed to distinguish between LNM-positive and LNM-negative patients and establish the optimal cut-off value. The sensitivity and specificity for each point could be obtained in ROC curve analysis, and the cut-off value was determined based on the point that maximized the sum of sensitivity and specificity. Univariate analysis involved the application of the chi-square test or Fisher’s exact test. The variables that showed statistical significance in the univariate analysis were then included in a multivariate logistic regression analysis to determine the independent risk factors for LNM. For the purpose of survival analysis, the log-rank test was employed. To identify prognostic factors, both univariate and multivariate Cox proportional hazards models were utilized. All *p*-values were two-sided, and *p*< 0.05 was deemed statistically significant.

## Results

### The proportions of lymphocyte subsets according to LNM status

**Figure 2:**
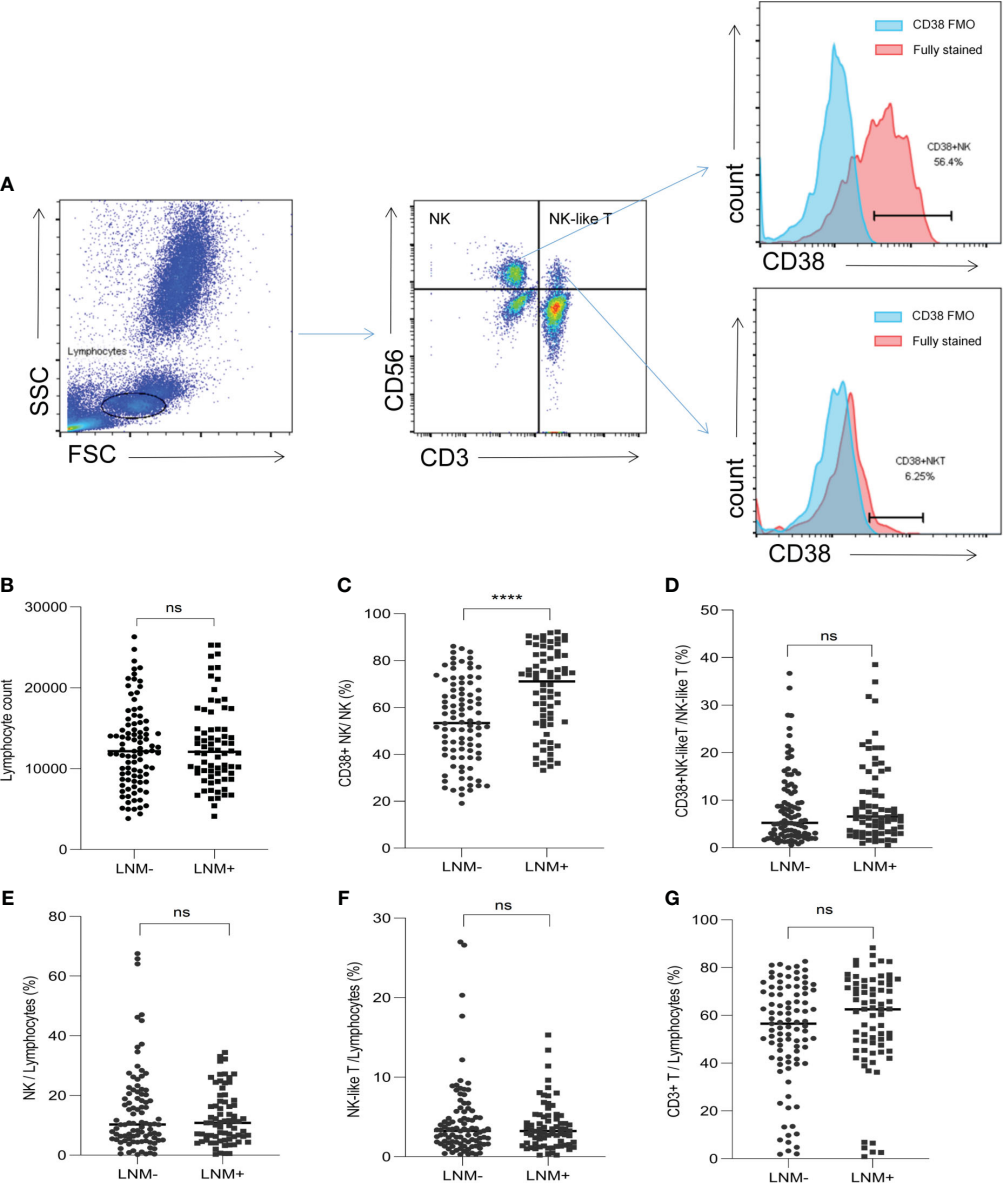
The proportions of lymphocyte subsets in CRC patients according to LNM status. **(A)** Flow cytometry plot showed the gating strategy for NK cells (CD3- CD56+), NK-like T cells (CD3+ CD56+), T cells (CD3+), CD38+ NK cells (CD3- CD56+ CD38+) and CD38+ NK-like T cells (CD3+ CD56+ CD38+). **(B)** The lymphocyte counts in LNM-positive and LNM-negative group. **(C)** The proportions of CD38+ NK cells to NK cells according to LNM; **(D)** The proportions of CD38+ NK-like T cells to NK-like T cells according to LNM; The proportion of NK cells **(E)**, NK-like T cells **(F)** and T cells **(G)** to lymphocytes according to LNM status. CRC, colorectal cancer; LNM, lymph node metastasis; CD38 FMO: CD38 fluorescence-minus-one; LNM+, patients with lymph node metastasis; LNM-, patients without lymph node metastasis. ns, not significant, ****: *p <*0. 0001.

The proportions of lymphocyte subsets in the peripheral blood of CRC patients were detected by flow cytometry. The representative flow cytometry plot was shown in **Figure 2A**. There was no difference in lymphocyte counts between the LNM-positive and LNM-negative groups (**Figure 2B**). In CRC patients with LNM, the proportion of CD38+ NK cells to NK cells was significantly increased (*p* < 0.0001) (**Figure 2C**). However, in the proportion of CD38+ NK-like T cells to total NK-like T cells, no difference was observed between patients with LNM and those without (**Figure 2D**). In addition, the proportions of NK cells (CD3- CD56+), NK-like T cells (CD3+ CD56+) and CD3+ T cells to lymphocytes showed no significant difference between the two groups (**Figures 2E–G**).

Further, the proportion of CD38+ CD56^bright^ and CD38+ CD56^dim^ NK cells between LNM-positive and LNM-negative patients were compared. The gating strategy for NK cell subtypes was shown in **Supplementary Figure 1A**. The proportions of CD38+ CD56^bright^ (*p*<0.0001) and CD38+ CD56^dim^ (*p*<0.0001) cells were increased in LNM-positive patients (**Supplementary Figures 1B, C**); And, the proportion of CD38 frequencies between CD56^bright^ and CD56^dim^ fractions showed no significant difference (*p*=0.11) (**Supplementary Figure 1D**).

**Figure 3:**
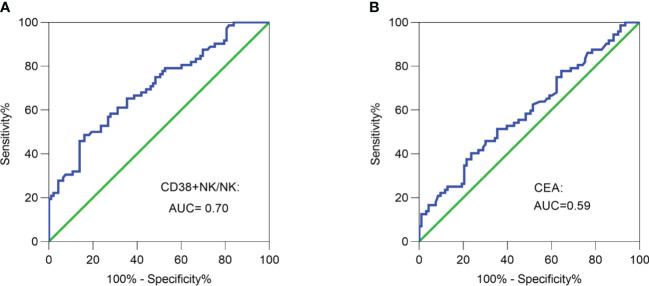
Receiver operating characteristic (ROC) curve analysis. **(A)** ROC curve analysis of the proportion of CD38+ NK cells was performed to differentiate LNM-positive patients and LNM-negative patients. **(B)** ROC curve analysis of CEA was performed to differentiate LNM-positive patients and LNM-negative patients. CRC, colorectal cancer; LNM, lymph node metastasis; CD38+ NK/NK, the proportions of CD38+ NK cells to NK cells; CEA, carcinoembryonic antigen.

ROC curve analysis was employed to differentiate LNM-positive patients and LNM-negative patients. The area under the curve (AUC) was determined to be 0.70, with a cut-off value of 72.85% (**Figure 3A**). The proportion of CD38+ NK cells had a superior ability in evaluating lymphnode status compared to CEA (AUC = 0.59) (**Figure 3B**).

### Clinical and pathological features of the CRC patients included in this study

The incidence of LNM in patients with tumor size ≥ 3 cm was 49.25% (66/134), which was significantly higher than in patients with tumor size < 3 cm (19.35%, 6/31) (*p* < 0.01). In terms of histologic grade, there was a higher incidence of LNM in patients with G2 (*p* < 0.01) compared to those with G1. Regarding depth of invasion, LNM was found in 19.44% (7/36) of the patients with T1/T2 stage, whereas 50.391% (65/129) of the patients with T3/T4 stage were LNM-positive (*p <*0.01). The incidence of LNM in patients with LVI was higher than those without (*p* < 0.01). The patients with perineural invasion demonstrated an increased likelihood of LNM (*p <*0.01). And, patients with CEA>5ng/ml had a higher incidence of LNM (*p* = 0.04). Considering that neoadjuvant therapy might affect the LNM status of patients, we included it in this analysis. The incidence of LNM in patients who did not receive neoadjuvant therapy was 45.81% (71/155), which was significantly higher than those recdeived (10.00%, 1/10) (*p* =0.03). Based on the results depicted in **Figure 2**, we included the proportion of CD38+ NK cells in the analysis of risk factors for LNM. And high proportion of CD38+ NK cells were associated with the incidence of LNM (*p <*0.01). No significant differences was observed in terms of age, sex, BMI, presence of ulcer and tumor location between LNM-positive patients and LNM-negative patients. The detailed results were shown in **Table 1**. Additionally, we conducted separate analyses on the relationship between LNM and clinicopathological factors in colon cancer and rectal cancer. The results showed the LNM in both colon cancer and rectal cancer was associated with tumor size, histologic grade, depth of invasion, LVI, perineural invasion, CEA, and CD38+ NK cells; Besides, LNM in rectal cancer was also associated with neoadjuvant therapy (**Supplementary Table 1**).

**Table 1:** Univariate analysis of risk factors for LNM in CRC.

	n (%)	Node negative, n (%)	Node positive, n (%)	*p* value
**Total**	165 (100.00)	93 (56.36)	72 (43.64)	
**Gender**				0.35
female	45 (27.27)	28 (16.97)	17 (10.30)	
male	120 (72.73)	65 (39.39)	55(33.33)	
**Age**				0.12
≤60	58 (35.15)	28 (16.97)	30 (18.18)	
>60	107 (64.85)	65 (39.39)	42 (25.46)	
**BMI**				0.60
≤28	149 (90.30)	83 (50.30)	66 (40.00)	
>28	16 (9.70)	10 (6.06)	6 (3.64)	
**Location**				0.17
colon	81 (49.09)	50 (30.30)	31 (18.79)	
rectum	84 (50.91)	43 (26.06)	41 (24.85)	
**Ulcer**				0.27
yes	131 (79.39)	71 (43.04)	60 (36.36)	
no	34 (20.61)	22 (13.33)	12 (7.27)	
**Tumor size**				<0.01
<3cm	31 (18.79)	25 (15.15)	6 (3.64)	
≥3cm	134 (81.21)	68 (41.21)	66 (40.00)	
**Histologic grade**				<0.01
G1	145 (87.88)	88 (53.33)	57 (34.55)	
G2	20 (12.12)	5 (3.03)	15 (9.09)	
**Depth of invasion**				<0.01
T1-T2	36 (21.82)	29 (17.58)	7 (4.24)	
T3-T4	129 (78.18)	64 (38.79)	65 (39.39)	
**LVI**				<0.01
yes	30 (18.18)	2 (1.21)	28 (16.97)	
no	135 (81.82)	91 (55.15)	44 (26.67)	
**Perineural invasion**				<0.01
yes	53 (32.12)	21 (12.73)	32 (19.39)	
no	112 (67.88)	72 (43.64)	40 (24.24)	
**CEA (ng/ml)**				0.04
≥ 5	48 (29.09)	21 (12.73)	27 (16.36)	
< 5	117 (70.91)	72 (43.64)	45 (27.27)	
neoadjuvant therapy				
yes	10 (6.06)	9 (5.45)	1 (0.61)	0.03
no	155 (93.94)	84 (50.91)	71 (43.03)	
**CD38+ NK cells**				<0.01
low (<cutoff)	115 (69.70)	78 (42.27)	37 (22.42)	
high (≥cutoff)	50 (30.30)	15 (9.09)	35 (21.21)	

LNM, lymph node metastasis; CRC, colorectal cancer; Histologic grade: G1, well or moderately differentiated adenocarcinomas; G2, poorly differentiated adenocarcinomas or signet ring cell type or mucinous carcinomas; LVI, lymphovascular invasion.

### Risk factors associated with the LNM in CRC patients

The univariate analysis revealed that several factors were associated with LNM, including histologic grade (G2), tumor size (≥3cm), depth of invasion (T3-T4), perineural invasion, LVI, CEA and high proportion of CD38+ NK cells. Subsequent stepwise logistic analysis revealed independent risk factors for LNM in CRC. These factors included histologic grade (G2) (OR = 4.76, *p* = 0.03), LVI (OR = 22.38, *p* < 0.01), and high proportion of CD38+ NK cells (OR = 4.54, *p* < 0.01). Multivariate analysis demonstrated neoadjuvant therapy tended to be a protective factor against LNM (OR=0.12), but this finding was not statistically significant (*p* = 0.11). The detailed results of the multivariate analysis were provided in **Table 2**.

**Table 2:** Multivariate logistic regression analysis of LNM in CRC.

	Odds ratio	95% CI	*p* value
**Depth of invasion (T3-T4)**	2.63	0.87-7.92	0.09
**Histologic grade (G2)**	4.76	1.25-18.07	0.03
**Tumor size (≥3cm)**	2.04	0.61-6.82	0.25
**CEA (>5ng/ml)**	1.19	0.50-2.84	0.70
**LVI**	22.38	3.97-104.68	<0.01
**Perineural invasion**	1.39	0.641-3.20	0.44
**neoadjuvant therapy (yes)**	0.12	0.01-1.63	0.11
**CD38+ NK cells (high)**	4.54	1.92-10.76	<0.01

LNM, lymph node metastasis; CRC, colorectal cancer; Histologic grade (G2), poorly differentiated adenocarcinomas or signet ring cell type or mucinous carcinomas; LVI, lymphovascular invasion.

### The relationship between CD38+ NK cells and prognosis in CRC patients

**Figure 4:**
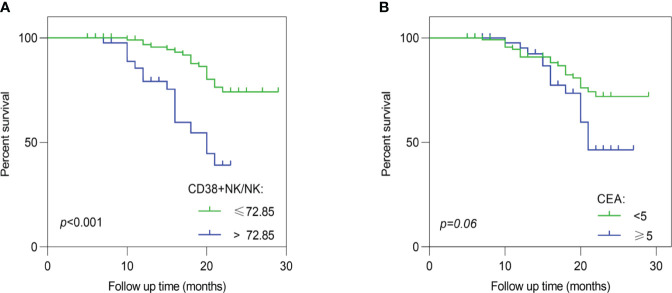
Log-rank analysis about the proportion of CD38+ NK cells and CEA. **(A)** The proportion of CD38+ NK cells was associated with poor prognosis of CRC patients. **(B)** No significant correlation was observed between serum CEA levels and prognosis of CRC. CRC, colorectal cancer; CD38+ NK/NK, the proportions of CD38+ NK cells to NK cells; CEA, carcinoembryonic antigen.

The survival status of the CRC patients in this study was obtained from hospitalization records and telephone follow-up. The survival analysis demonstrated a significant association between the proportion of CD38+ NK cells to NK cells and poor prognosis in CRC patients (*p* < 0.001), as depicted in **Figure 4A**. The capability of CD38+ NK cells to evaluate prognosis was superior to CEA (*p* = 0.06) (**Figure 4B**). Furthermore, we investigated the relationship between clinical pathological factors and prognosis in CRC patients. Following both univariate and multivariate Cox regression analyses, the histologic grade (G2) (HR = 3.14; *p* = 0.01), LVI (HR = 3.98; *p* < 0.01), and higher proportion of CD38+ NK cells (HR = 2.37; *p* = 0.03) were identified as independent risk factors for poor prognosis of CRC. The details were shown in **Table 3**.

**Table 3:** Cox-regression analysis of the risk factors for poor prognosis in CRC.

	Univariate			Multivariate		
	HR	95%CI	*p* value	HR	95%CI	*p* value
**Gender (male)**	0.62	0.31-1.22	0.16			
**Age (≥60)**	0.81	0.41-1.59	0.54			
**BMI (>28)**	1.65	0.63-4.27	0.31			
**Location (rectum)**	0.68	0.34-1.33	0.26			
**Ulcer (yes)**	2.42	0.85-6.88	0.10			
**CEA (≥ 5ng/ml)**	1.87	0.94-3.71	0.08			
**Tumor size (≥3cm)**	9.00	1.23-65.88	0.03	7.49	0.96-58.50	0.06
**Histologic grade (G2)**	2.68	1.25-5.74	0.01	3.14	1.36-7.26	0.01
**Depth of invasion (T3-T4)**	4.40	1.34-14.42	0.01	1.42	0.39-5.19	0.60
**LVI**	7.22	3.62-14.37	<0.01	3.98	1.65-9.61	<0.01
**Perineural invasion**	2.70	1.37-5.32	<0.01	1.44	0.65-3.17	0.37
**CD38+ NK cells (high)**	3.77	1.91-7.44	<0.01	2.37	1.11-5.05	0.03

CRC, colorectal cancer; Histologic grade (G2), poorly differentiated adenocarcinomas or signet ring cell type or mucinous carcinomas; LVI, lymphovascular invasion.

### CD38+ NK cells were overexpressed in early-stage CRC with LNM compared to those without

**Figure 5:**
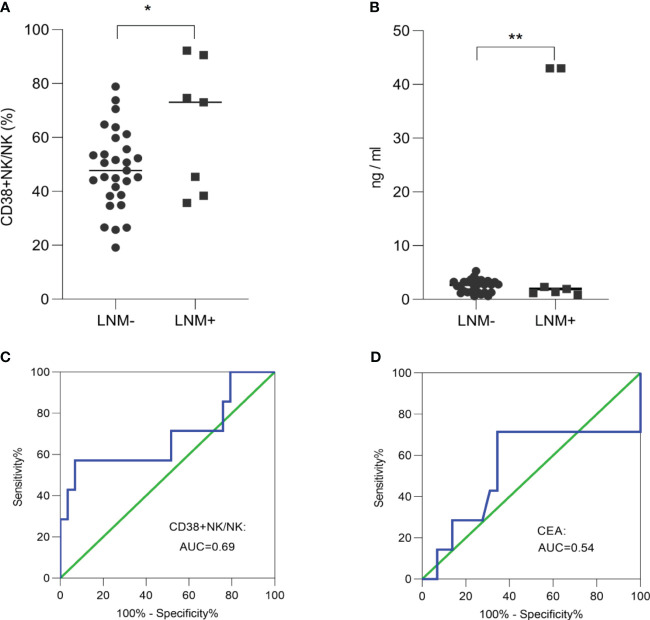
The ability of CD38+ NK cells and CEA to evaluate lymph node status in early-stage CRC. The proportion of CD38+ NK cells to NK cells **(A)** and the levels of CEA **(B)** was elevated in early-stage CRC patients with LNM. **(C)** ROC curve analysis of the proportion of CD38+ NK cells was performed to distinguish between early-stage CRC patients with LNM and those without LNM. **(D)** ROC curve analysis of CEA was performed to distinguish between early-stage CRC patients with LNM and those without LNM. CRC, colorectal cancer; LNM, lymph node metastasis; CD38+ NK/NK, the proportions of CD38+ NK cells to NK cells; CEA, carcinoembryonic antigen. *: *p <*0.05; **: *p <*0.01.

In order to investigate the ability of CD38+ NK cells and CEA to evaluate lymph node status in early-stage CRC, we compared the proportions of CD38+ NK cells between T1/T2-stage patients with LNM and those without. The proportion of CD38+ NK cells to NK cells was significantly increased in LNM-positive group (*p* = 0.03 (**Figure 5A**). The levels of CEA was also elevated in CRC patients with LNM (*p* = 0.005) (**Figure 5B**). The ROC curve analysis showed that the AUC for the proportion of CD38+ NK cells was 0.69 (**Figure 5C**), while the AUC for CEA was 0.54 (**Figure 5D**).

## Discussion

In humans, NK cells are a widely distributed type of innate lymphoid cells, which specifically expresses CD56 but not CD3 (6). These cells play a significant role in the immune response against viral infections and tumors by releasing cytokines, chemokines, and displaying cytotoxic abilities (6). Previous studies had reported that NK cells play a significant role in the diagnosis and prognosis of coronary artery disease (10), papillary thyroid cancer (7), and CRC (11, 12). CD38 was expressed on the surface of NK cells (15); And CD38+ NK cell was a subtype of NK cells. Morandi et al. (13) demonstrated that CD38+ NK cells could exert immunosuppressive effects by inhibiting the proliferation of CD4+ T cells. Our research group has found that CD38+ NK cells in the peripheral blood of rheumatoid arthritis (RA) patients play an important role in regulating immune balance (16). In this study, we found that the proportion of CD38+ NK cells was associated with LNM and it was an independent risk factor for LNM and poor prognosis in CRC patients.

We investigated the proportion of lymphocyte subsets in the peripheral blood of CRC patients. The results showed there was no significant difference in the proportion of NK cells between LNM-positive and LNM-negative patients. Krijgsman, et al. (17) reported that the proportion of NK cells in the blood of CRC patients did not differ significantly from that of healthy donors. However, in CRC tumor microenvironment, NK cells were generally scarce (18–20), which might be related to the impaired migration of NK cells into CRC tumor tissue (18). Further research is warranted to investigate the disparities in expression and functionality of NK cells between peripheral blood and the tumor microenvironment.

Among the lymphocyte subsets measured in this study, the proportion of CD38+ NK cells to NK cells was increased in CRC patients with LNM. And, the proportion of CD38 frequencies between CD56^bright^ and CD56^dim^ fractions showed no significant difference. Additionally, the elevated proportion of CD38+ NK cells was associated with adverse outcomes of CRC. Moreover, it served as an independent risk factor for both LNM and poor prognosis. In tumor cells, CD38 had been shown to facilitate tumor proliferation and migration in lung and cervical cancer (21, 22). Additionally, Deckert et al. (23) discovered that inhibiting CD38 in multiple myeloma cells promoted cellular apoptosis. As widely recognized, CD38 is an enzyme responsible for the hydrolysis of nicotinamide adenine dinucleotide (NAD+), leading to the generation of adenosine diphosphate ribose (ADPR) or cyclic ADPR (cADPR) (24–26). This enzymatic activity has been shown to impact calcium signaling and release, resulting in the decrease of extracellular NAD+ levels, alteration of calcium cascade reactions, and facilitation of adenosine-mediated immune suppression (24–26). In immune cells, inhibiting CD38 expression on T cells enhanced their anti-tumor effect through the CD38-NAD+ axis. In addition, CD38KO NK cells produced higher amounts of IFN-γ production and exhibited enhanced anti-tumor activity (27). In addition, Wang et al. (16) found that CD38 on NK cells could modulate T cell immune balance by regulating cytokine secretion.

Further, the previous studies had proposed that the impact of CD38 on immune cells is primarily mediated through the regulation of FasL expression (28); Alterations in CD38/FasL-mediated NK cell apoptosis have been reported in gastric cancer (29). These evidences emphasized the role of CD38 in malignant tumors and underscored its significance as a target in cancer immunotherapy (30).

CEA was commonly used as the primary marker for diagnosis and monitor of CRC. The levels of CEA in serum could also be used as an indicator to assess LNM in CRC (31), but its effectiveness was not optima (32). Our results demonstrated that the proportion of CD38+ NK cells had a superior ability in evaluating lymph node status and prognosis of CRC compared to CEA. Cells that co-express CD3 and CD56 are referred to as NK-like T cells (33, 34). NK-like T cells possess cytotoxicity but are primarily recognized for their significant regulatory functions, amplifying or suppressing immune responses through the secretion of abundant pro-inflammatory or anti-inflammatory cytokines upon activation (35). Zhou et al. (36) found that an elevated level of CD3+ CD56+ CD16+ NK-like T cells was associated with increased pregnancy rates and live birth rates in *in vitro* fertilization treatment. Krijgsman et al. (17) pointed out that CD3+ CD56+ NK-like T cells in CRC patients were not associated with disease-free survival, which was consistent with our research findings. Furthermore, this study demonstrated that there was no significant difference in the proportion of CD38+ NK-like T cells between CRC patients with and without LNM.

Regarding the assessment of clinicopathologic factors, the findings of this study indicated that the poorer histologic grade and LVI were independent risk factors for LNM in CRC patients; Additionally, it was found the poorer histologic grade and presence of LVI were also associated with adverse prognosis. These findings were in concordance with previous research (37, 38). Tumor size and depth of invasion were sometimes considered risk factors for LNM, but in this study, independent risk factors for LNM did not include either. Indeed, there were studies reported that tumor size was not correlated with LNM (39, 40). Additionally, previous studies found the depth of invasion was not associated with LNM (4, 40, 41). These were consistent with our findings. The differing critical thresholds for tumor size, inconsistent diagnostic criteria for invasion depth, and individual variations among clinical doctors might contribute to the disparate outcomes. Additionally, in univariate analysis, neoadjuvant therapy was associated with LNM in CRC patients. However, multivariate analysis suggested that neoadjuvant therapy was not a protective factor against LNM in CRC patients. The proportion of rectal cancer patients receiving neoadjuvant therapy in this study was relatively low. This might be attributed to a relatively higher proportion of early-stage rectal cancer in the collected samples, discrepancies between clinical staging and pathological staging, local symptoms such as bleeding and obstruction, and patient or family refusal of neoadjuvant therapy. Further studies can be conducted by expanding the collection of patients who have received neoadjuvant therapy to investigate the effects of neoadjuvant therapy on LNM in CRC, especially in rectal cancer patients.

According to the latest guidelines for CRC treatment (3), the JSCCR stated that endoscopic resection is recommended for cases of intramucosal carcinoma or slight submucosal carcinoma with negligible risk of LNM. Therefore, assessing LNM in early-stage CRC becomes crucial in guiding clinical decision-making. This study revealed that early-stage CRC patients with LNM had a higher proportion of CD38+ NK cells, suggesting that the CD38+ NK cells was expected to be a potential marker for LNM in early-stage CRC. ROC curve analysis showed the ability of the proportion of CD38+ NK cells to evaluate lymph node status in early-stage CRC outperformed CEA. However, the analysis conducted in this study lacked in-depth detail due to the small sample size of early-stage patients. It will be crucial for future studies to employ larger sample sizes and continue with long-term follow-ups to obtain more detailed results. Indeed, the samples enrolled in this study were screened by rigorous inclusion and exclusion criteria, indicating the results might be reproducible and reliable. Due to the potential impact of chemotherapy on the immune system, which might affect the levels of immune cells (12), we had collected pre-treatment samples in this study. In the subsequent phase, we will further enrich this data set and analyze the effects of chemotherapy on the proportion of lymphocyte subsets. In addition, the functional mechanism of CD38+ NK cells was not extensively investigated in this study. Our research group had previously reported CD38+ NK cells in blood and synovial fluids mediated T-cell immune imbalance in RA, whereas CD38- NK cells had no such role (16). Further exploration can be conducted on the role of CD38+ NK cells in modulating T-cell immune balance in blood and tumor microenvironment (TME) of CRC patients. Moreover, CD38 mediated the production of adenosine under the synergistic action of CD203a and CDD73, thereby exerting immunosuppressive effects (42–44). Analyzing the expression of these molecules in NK cells might serve as a promising strategy to unravel the underlying mechanisms of CD38+ NK cell functionality.

## Conclusions

The proportion of CD38+ NK cells to total NK cells was increased in CRC patients with LNM compared to those without. A high proportion of CD38+ NK cells is an independent risk factor for LNM, and it is associated with poor prognosis in CRC patients. The proportion of CD38+ NK cells is expected to be a promising marker for LNM and prognosis in patients with CRC before initial treatment.

## Data availability statement

The original contributions presented in the study are included in the article/**Supplementary Material**. Further inquiries can be directed to the corresponding authors.

## Ethics statement

The studies involving humans were approved by The Ethics Committee of the Affiliated Hospital of Qingdao University (QYFYWZLL28002). The studies were conducted in accordance with the local legislation and institutional requirements. The participants provided their written informed consent to participate in this study.

## Author contributions

XW: Conceptualization, Investigation, Methodology, Project administration, Software, Writing – original draft, Writing – review & editing. HL: Data curation, Validation, Writing – review & editing. HC: Formal analysis, Writing – review & editing. KF: Resources, Supervision, Writing – review & editing. XC: Conceptualization, Funding acquisition, Visualization, Writing – review & editing.
