# Supplementary material for: Overexpression of circulating CD38+ NK cells in colorectal cancer was associated with lymph node metastasis and poor prognosis
Source: Front Oncol. 2024 Feb 23;14:1309785. doi: 10.3389/fonc.2024.1309785 (PMC10921414; doi:10.3389/fonc.2024.1309785)
Supplement: Supplementary file 1 [file DataSheet_1.pdf]

Supplementary Material

1. Figure

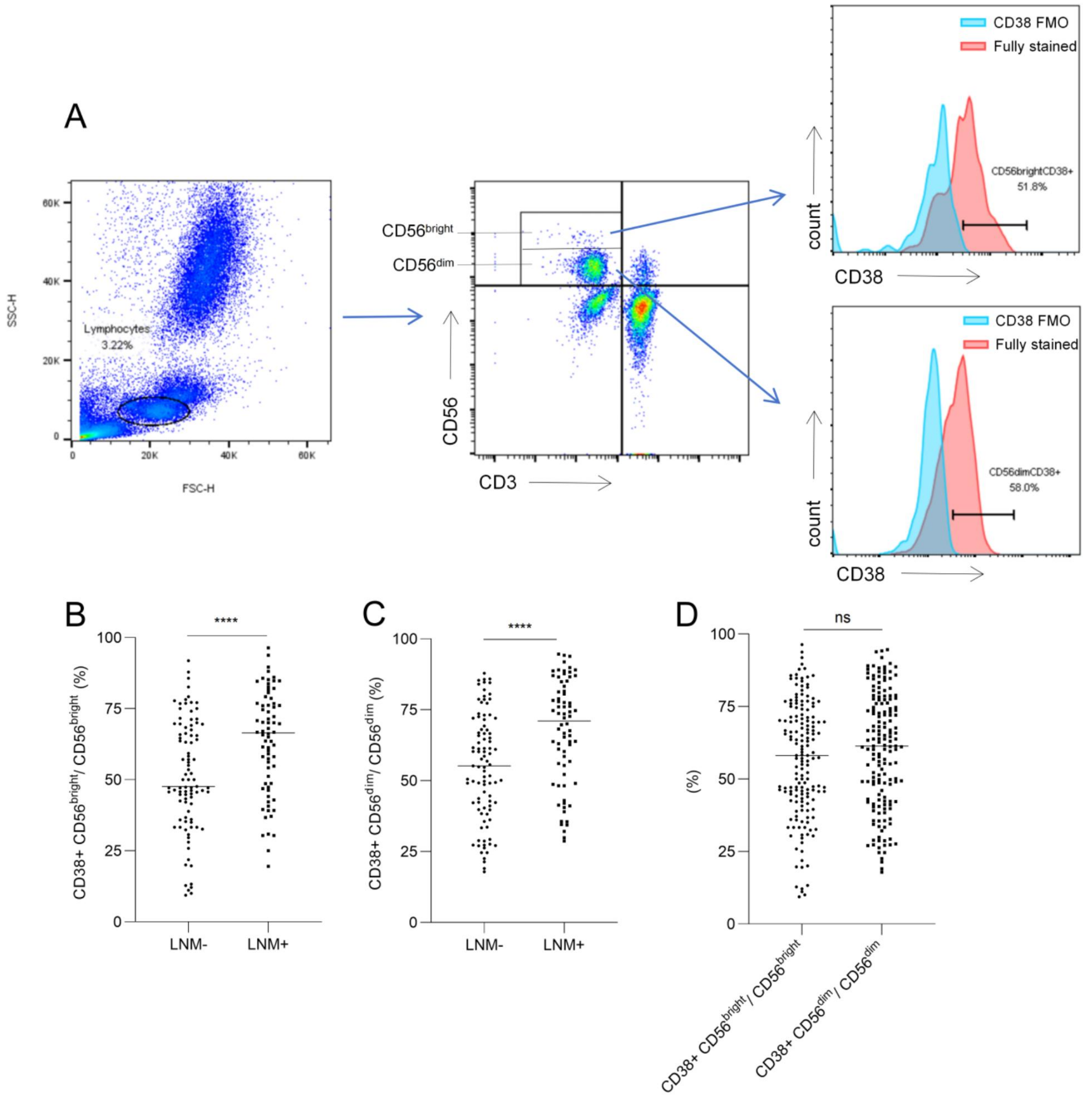

Figure S1. The proportions of NK cell subtype in CRC patients. **(A)** Flow cytometry plot showed the gating strategy for CD56^{bright} NK cells, CD56^{dim} NK cells, CD38+ CD56^{bright} NK cells and CD38+ CD56^{dim} NK cells. **(B)** The proportions of CD38+ CD56^{bright} NK cells to CD56^{bright} NK cells according to LNM; **(C)** The proportions of CD38+ CD56^{dim} NK cells to CD56^{dim} NK cells according to LNM; **(D)** The proportion of CD38 frequencies in CD56bright and CD56dim fractions. CRC, colorectal cancer; LNM, lymph node metastasis; CD38 FMO: CD38 fluorescence-minus-one; LNM+: patients with lymph node metastasis; LNM-: patients without lymph node metastasis. ns: not significant, ****: $p \leqslant 0.0001$.

2. Table

Table S1. The relationship between lymph node metastasis and clinicopathological factors in colon cancer and rectal cancer.

	Colon, n(%)				Rectum, n(%)			
	**Total**	**LNM+**	**LNM-**	***p***	**Total**	**LNM+**	**LNM-**	***p***
**Total**	81 (100.00)	31 (38.27)	50 (61.73)		84 (100.00)	41 (48.81)	43 (51.19)	
**Gender**				0.54				0.07
female	28 (34.57)	12 (14.81)	16 (19.75)		17 (20.24)	5 (5.95)	12 (14.29)	
male	53 (65.43)	19 (23.46)	34 (41.98)		67 (79.76)	36 (42.86)	31 (36.90)	
**Age**				0.14				0.26
≤60	36 (44.44)	17 (20.99)	19 (23.46)		22 (26.19)	13 (15.48)	9 (10.71)	
>60	45 (55.56)	14 (17.28)	31 (38.27)		62 (73.81)	28 (33.33)	34 (40.48)	
**BMI**				0.31				0.28
≤28	69 (85.19)	28 (34.57)	41 (50.62)		80 (95.24)	38 (45.24)	42 (50.00)	
>28	12(14.81)	3 (3.70)	9 (11.11)		4 (4.76)	3 (3.57)	1 (1.19)	
**Ulcer**				0.78				0.21
no	17 (20.99)	6 (7.41)	11 (13.58)		17 (20.24)	6 (7.14)	11 (13.10)	
yes	64 (79.01)	25 (30.86)	39 (48.15)		67 (79.76)	35 (41.67)	32 (38.10)	
**Tumor size**				0.04				0.02
<3cm	14 (17.28)	2 (2.47)	12 (14.81)		17 (20.24)	4 (4.76)	13 (15.48)	
≥3cm	67 (82.72)	29 (35.80)	38 (46.91)		67 (79.76)	37 (44.05)	30 (35.71)	
**Histologic grade**				0.03				0.02
G1	69 (85.19)	23 (28.40)	46 (56.79)		76 (90.48)	34 (40.48)	42 (50.00)	
G2	12 (14.81)	8 (9.88)	4 (4.94)		8 (9.52)	7 (8.33)	1 (1.19)	
**Depth of invasion**				0.04				<0.01
T1-T2	10 (12.35)	1 (1.23)	9 (11.11)		26 (30.95)	6 (7.14)	20 (23.81)	
T3-T4	71 (87.65)	30 (37.04)	41 (50.62)		58 (69.05)	35 (41.67)	23 (27.38)	
**LVI**				<0.01				<0.01
yes	18 (22.22)	16 (19.75)	2 (2.47)		12 (14.29)	12 (14.29)	0 (0.00)	
no	63 (77.78)	15 (18.52)	48 (59.26)		72 (85.71)	29 (34.52)	43 (51.19)	
**Perineural invasion**				0.04				0.04
yes	21 (25.93)	12 (14.81)	9 (11.11)		32 (38.10)	20 (23.81)	12 (14.29)	
no	60 (74.07)	19 (23.46)	41 (50.62)		52 (61.90)	21 (25.00)	31 (36.90)	
**CEA (ng/ml)**				0.03				<0.01
≥ 5	30 (37.04)	16 (19.75)	14 (17.28)		18 (21.43)	11 (13.10)	7 (8.33)	
< 5	51 (62.96)	15 (18.52)	36 (44.44)		66 (78.57)	30 (35.71)	36 (42.86)	
**neoadjuvant therapy**				0.26				0.03
yes	2 (2.47)	0 (0.00)	2 (2.47)		8 (9.52)	1 (1.19)	7 (8.33)	
no	79 (97.53)	31 (38.27)	48 (59.26)		76 (90.48)	40 (47.62)	36 (42.86)	
**CD38+ NK cells**				0.02				<0.01
low (<cutoff)	59 (72.84)	18 (22.22)	41 (50.62)		56 (66.67)	19 (22.62)	37 (44.05)	
high (≥cutoff)	22 (27.16)	13 (16.05)	9 (11.11)		28 (33.33)	22 (26.19)	6 (7.14)	

LNM, lymph node metastasis; CRC, colorectal cancer; Histologic grade: G1, well or moderately differentiated adenocarcinomas; G2, poorly differentiated adenocarcinomas or signet ring cell type or mucinous carcinomas; LVI, lymphovascular invasion.
